# Coping with Stress in Athletes: Psychological Strategies and Psychometric Tools for Stress and Anxiety Assessment

**DOI:** 10.3390/jcm15176582

**Published:** 2026-08-26

**Authors:** Franciszek Kaczmarek, Monika Matecka, Joanna Bartkowiak-Wieczorek, Michał Kmiecik, Katarzyna Kaczmarek, Paulina Gajniak, Mateusz Fiutak, Joanna Stężycka, Kamil Krzysztof Krupa, Sonia Marchwiak, Michał Nowak, Michalina Cuper, Paulina Cuper, Karolina Jenczylik, Przemysław Paterek, Edyta Mądry

**Affiliations:** 1 Franciszek Raszeja Municipal Hospital, 60-834 Poznań, Poland; franciszek.kaczmarek@pm.me (F.K.); kmiecik.michal@proton.me (M.K.); 2 Department of Occupational Therapy, Poznań University of Medical Sciences, 61-701 Poznań, Poland; mmatecka@ump.edu.pl; 3 Department of Physiology, Poznań University of Medical Sciences, 60-179 Poznań, Poland; emadry@ump.edu.pl; 4 Faculty of Psychology and Law, SWPS University, 61-719 Poznań, Poland; kaczmarek.katarzyna.swps@pm.me (K.K.); flow4_gojenczylik@op.pl (K.J.); 5 J. Struś Municipal Multidisciplinary Hospital, 61-285 Poznań, Poland; paulina.gajniak@interia.pl; 6 University Clinical Hospital of Poznań, Poznań University of Medical Sciences, 61-701 Poznań, Poland; mateusz.fiutak@usk.poznan.pl (M.F.); mn.nowak@pm.me (M.N.); 7 Student Scientific Society, Poznań University of Medical Sciences, 61-701 Poznań, Poland; 88871@student.ump.edu.pl (J.S.); 88992@student.ump.edu.pl (K.K.K.); 8 Voivodeship Hospital, 60-479 Poznań, Poland; sonix03marchwiak@wp.pl; 9 Medical University of Silesia, 41-808 Zabrze, Poland; michalina.cuper@op.pl; 10 Pleszew Medical Centre, 63-300 Pleszew, Poland; pa.cuper@proton.me (P.C.); przemek.paterek@vp.pl (P.P.)

**Keywords:** sport-related stress and anxiety, coping strategy, psychological stress assessment tools, psychological interventions in professional sport

## Abstract

**Background and objectives**: It is now increasingly acknowledged that psychological resilience and effective stress management are important factors in determining performance in professional sport. The present review examines various psychological interventions used in sport psychology, with particular emphasis on methods for improving performance under competitive pressure. It also assesses the psychometric scales and paradigm-based tools available for measuring stress, stress reactivity, and pre-competition anxiety in athletes. **Methods**: A narrative review was carried out using the OVID, EBSCO, PubMed, SCOPUS, Web of Science, and Google Scholar databases, with a preference for randomised controlled trials, observational studies, systematic reviews, meta-analyses, and consensus guidelines. **Results**: The results form the foundation for creating more thorough athlete support programmes. Psychological interventions are necessary to improve or maintain sporting performance, manage stress and anxiety, promote healthy sleep habits, and reduce the risk of injury. **Conclusions**: Athletic performance is influenced by technical, tactical, physical, and psychological factors. Psychological interventions, including self-talk, imagery, goal setting, mindfulness, and relaxation techniques, help manage stress while maintaining focus and motivation. It is recommended that athlete sleep education and psychosocially informed injury prevention be incorporated into these strategies.

## 1. Introduction

In sports competitions, athletes go through both positive and negative emotional states, mainly stress and anxiety. Stress can be evaluated subjectively using different tools and objectively using measurable data. Objective indicators involve checking blood pressure, heart rate, heart rate variability and biochemical markers such as cortisol. The experience of psychological stress may result in mental health problems, such as anxiety, depression and mental fatigue. The depressive process relates to hyperactivity or dysregulation of the hypothalamic–pituitary–adrenal (HPA) axis [1]. Chronic psychological stress may reduce athletes’ ability to meet the demands of their daily training and competitions, thus increasing the chance of them leaving the sport [2,3]. A variety of technical, tactical, physical, psychological and social factors influence sporting performance [4]. The results of competitions are closely linked to the individual cognitive, behavioural, and physiological reactions to stressful situations [5]. Athletes also must cope with additional risk factors which affect their mental health; competition can cause, worsen or bring forward psychological problems [6]. There is a strong connection between mental health and competitive performance [7]. As stated in the International Olympic Committee (IOC) consensus statement, “mental health is an integral part of the well-being and performance of elite athletes and must not be separated from physical health. Mental health assessment and management in elite athletes should be as common and as easily accessible as their other medical care; ideally elite athletes should have access to the best interdisciplinary care” [8]. Members of sports support staff (coaches, sports psychologists, medical personnel and managers) should realise that psychological, cultural and environmental factors influence the mental health and well-being of athletes. It is essential to identify and monitor risk factors early on, given their complex nature and how they interact, if comprehensive, integrated, and appropriate support is to be provided. Professional help is required in high-risk situations or at certain times such as after a serious injury, during the transition to retirement and following performance difficulties [3].

### 1.1. IOC Guidelines for Stress Management and Mental Health in Elite Athletes

The IOC considers stress an essential element of sport and views good mental health as a fundamental part of overall health. The main purpose of the IOC guidelines is to teach athletes how to manage stress, reduce its levels, support their mental health, and improve their stress tolerance. These matters are covered in important publications, including the IOC Consensus Statement on Mental Health in Elite Athletes (2019), the IOC Mental Health Action Plan (2023), and the Mental Health Guidelines for Major Sporting Events (2024) [8,9,10]. The guidelines state that effective stress management requires a thorough, integrated approach that involves developing individual psychological skills, creating supportive environments, and ensuring that professional care is available at all stages of the sporting cycle. They stress that good mental health is just as important as physical fitness, and that the combination of these two elements is key to achieving success in sport.

#### 1.1.1. Supporting Mental Health Literacy and Psycho-Educational Skills

The IOC emphasises the need to inform athletes and their support staff about the signs and symptoms of stress and to help them build mental skills for coping. This involves things such as mindfulness training, emotion regulation and reframing, all of which assist athletes in handling pressure and staying focused as they develop adaptive responses to adversity. Furthermore, by normalising conversations regarding mental health, the stigma associated with it is reduced, and early treatment is encouraged [8].

#### 1.1.2. Creating a Supportive and Safe Sporting Environment

The IOC suggests that the sporting environment be improved by reducing various stressors, such as too much travel, tight competition schedules, and inadequate recovery time. Safeguarding policies must ensure that athletes are protected against abuse, harassment, and other psychosocial risks. It is essential to provide conditions that promote rest, safety, and psychological comfort to achieve effective stress management [10].

#### 1.1.3. Cooperation with Qualified Mental Health Professionals

The following IOC recommendation relates to athletes working with mental health professionals as part of the sports support system. Such specialists ought to offer confidential and stigma-free services and should take part in both preventive and interventive care. It is recommended that standardised tools, for example the Sport Mental Health Assessment Tool 1 (SMHAT-1), be used to aid the early identification of stress-related problems and to help guide appropriate interventions [8,11].

#### 1.1.4. In-Competition Stress Management Interventions

Since major competitions are linked to increased stress, the IOC recommends putting in place specific types of support. This involves offering ‘mental fitness’ or recovery areas, ensuring that athletes have access to psychological support on site, and having clearly defined Mental Health Emergency Action Plans. These measures allow athletes to cope with acute stress and to get immediate help when it is needed [10].

#### 1.1.5. Post-Competition Psychological Support

The IOC points out the possibility of a ‘post-competition crash’, during which athletes might go through an emotional low after taking part in major events. To address this, it is advised to provide continuous psychological monitoring and support as they re-enter their usual training or everyday life. This is important for ensuring long-term mental health and for preventing delayed stress reactions [8,9].

#### 1.1.6. Monitoring of Psychological Load

The IOC suggests that athletes’ psychological load should be monitored systematically, just as physical load is tracked. This kind of monitoring would involve self-report well-being questionnaires, assessment of mental fatigue, and integration of psychological data into broader performance monitoring systems. With this method, it is possible to identify early on when there is excessive stress and to stop it from developing into more serious mental health problems [8].

#### 1.1.7. Individualised Approach

The IOC states that when formulating stress management strategies, it is necessary to take into account the specific needs and preferences of the individual athlete; personality factors, sport-specific factors, age, life and career stage, and country of origin should all be considered to make sure that the coping methods are both effective and meaningful [9].

#### 1.1.8. Integration of Mental Health into Sports Medicine

It would be better to regard psychological stress as belonging to the broader field of sports medicine rather than as a separate matter. Following best practice, the psychosocial team should work closely together, conduct joint interviews with the athletes, and evaluate mental stress and clinical symptoms in the same manner as physical injuries. This approach supports care that is centred on the athlete and helps to achieve more effective stress reduction [8].

#### 1.1.9. Mental Health Emergency Preparedness

The IOC also advises on how to deal with acute psychological crises, for example those which take place at major sporting events. The measures involved include a Mental Health Emergency Action Plan, quick access to qualified healthcare professionals, and escalation procedures. This is intended to ensure an immediate and effective response to cases such as panic attacks, episodes of distress, or mental health breakdowns [10].

#### 1.1.10. Dual Career and Life Balance

The IOC stresses the need to prevent an imbalance between sport and personal life and believes that by supporting athletes in their educational, vocational, and personal development, it is possible to reduce the psychological strain on them and promote their growth. This kind of support is especially important at times of transition, for example during retirement, since these periods are linked to a higher risk of stress [9].

#### 1.1.11. Sleep and Recovery as Stress Regulators

The IOC regards sleep and recovery as vital elements in stress management. Disruption to sleep and recovery caused by travel, the dynamics of competition, or environmental factors (e.g., jet lag) can lead to a significant increase in psychological stressors. Consequently, structured recovery and optimisation of sleep are critical to maintaining a healthy psychological state [10].

The key recommendations of the IOC regarding stress management among elite athletes are given in Table 1.

The aim of this narrative review is therefore to present selected psychological interventions used in sport psychology practice, with particular emphasis on approaches that support performance under competitive pressure, and to review the psychometric scales and paradigm-based tools available for assessing stress, stress reactivity and pre-competition anxiety in athletes.

## 2. Materials and Methods

A narrative review was carried out using the OVID, EBSCO, PubMed, SCOPUS (Elsevier), Web of Science, and Google Scholar databases. Full texts of the articles that had been identified through these databases were obtained via the databases themselves; where a full text was not accessible in this way, it was retrieved from ResearchGate, which was not used as a source for carrying out bibliographic searches. The literature search covered all publications up to June 2026, with no minimum date limit to include foundational studies relating to the development of psychological interventions and stress-assessment tools. The review was divided into three thematic sections: “Psychological interventions and relaxation techniques”, “Psychological stress assessment tools”, and “Coping strategies”. Each subsection was searched and screened independently by two reviewers. All six databases—PubMed, OVID, EBSCO, SCOPUS (Elsevier), Web of Science, and Google Scholar—were searched for every thematic concept block covering these three subsections; the block covering the IOC guidance and the background blocks for the Introduction and Discussion were approached differently, as described below.

For each subsection, topic-specific search strings were created by combining terms related to the intervention or tool with a block referring to the population or context, using Boolean operators. The following English terms and their combinations were included but not limited to: “progressive muscle relaxation”, “progressive muscle relaxation technique”, pmrt, “imagery and visualisation”, imagery, visualisation, “coping strategies”, “coping skills”, “coping mechanisms”, “coping behaviours”, self-talk, “inner speech”, “self-verbalisation”, “self-statements”, “internal dialogue”, “self-instruction”, “verbal self-guidance”, “relaxation techniques”, “relaxation training”, “relaxation therapy”, “relaxation response”, “relaxation intervention”, scale, questionnaire, inventory, test, tool, measure, athlete, athletes, sport, sports, athletics, performance, player, players, elite, “elite athletes”, “competitive athlete”, sportswoman, sportsman, competitive, “Athlete Psychological Strain Questionnaire”, APSQ, “Competitive State Anxiety Inventory”, CSAI, “Sport Anxiety Scale”, SAS, “Sport Competition Anxiety Test”, SCAT, “State-Trait Anxiety Inventory”, STAI, “Trier Social Stress Test”, TSST, “Perceived Stress Reactivity Scale”, PSRS, “Profile of Mood States”, POMS, sleep, “sleep quality”, “sleep extension”, injury, “injury risk”, “injury prevention”, depression, “depressive symptoms”, burnout, resilience, cortisol. The search strategy also included grammatical and morphological variations in the selected keywords, such as singular and plural forms, alternative spellings, and related word combinations. Since each database has its own search capabilities, search strategies were tailored to use the appropriate filters.

Operationally, the search was based on 15 concept blocks that correspond to the structure of this review: one block dealing with the IOC’s guidance on mental health and stress management in elite sport, three blocks on psychological interventions and relaxation techniques, eight blocks on psychometric and paradigm-based assessment tools, one block on coping frameworks, and two blocks on background material for the Introduction and Discussion. Each database was searched using search terms consistent with the given concept block, so that each query corresponded to exactly one block of the review. The twelve thematic blocks—the three covering the psychological interventions and relaxation techniques, the eight covering the psychological stress assessment tools and the one covering coping frameworks—were each run across the six databases listed above, which together with the remaining targeted searches, yielded approximately 72 individual database queries. The record counts reported below are cumulative totals across all six databases; they sum the results returned in PubMed, OVID, EBSCO, SCOPUS (Elsevier), Web of Science, and Google Scholar. The block covering the IOC guidance and the two blocks covering background material for the Introduction and Discussion were not run systematically across all six databases. For the IOC block, the relevant documents were identified primarily from the official IOC documents repository (https://www.olympics.com/ioc/documents (accessed on 21 March 2026)), supplemented by targeted database searches, and were limited to the IOC consensus statements and guidelines on mental health and stress management in elite sport, as well as materials directly related to them. The two background blocks were searched mainly in PubMed, as described below. The final search was conducted across all six databases on 30 July 2026 and covered publications indexed through 30 June 2026.

To improve the transparency of the selection process, the overall flow of records can be summarised as follows. The focused concept blocks—those covering the psychological interventions and relaxation techniques, the psychological stress assessment tools and the coping frameworks, together with the block covering the IOC guidance, for which the selected documents drew primarily on official IOC materials—returned a total of 23,167 unique records across the six databases. After title and abstract screening and full-text assessment against the predefined inclusion and exclusion criteria described below, 120 publications were selected and are described in the thematic sections listed above.

Records were screened by title and abstract for alignment with the scope of the corresponding concept block. For any individual database query that returned more than 3000 records, the first 3000 results, in the order returned by the database, were read at the title and abstract level; for all remaining queries, the complete list of results was screened in full. Potentially relevant articles were assessed in full text against predefined inclusion and exclusion criteria. Priority was given to randomised controlled trials (RCTs), observational studies, systematic reviews and meta-analyses, and consensus guidelines. Opinion pieces, conference papers, and preprints were excluded. No language restriction was applied at screening, provided an English title and abstract were available; full texts of eligible articles in languages other than English or Polish were machine-translated (DeepL SE. (2026). DeepL Translator [https://www.deepl.com]) and assessed in full. Any disagreements between the two reviewers regarding study eligibility were resolved by consensus: the contested records were re-examined jointly against the predefined inclusion and exclusion criteria until agreement was reached.

The two remaining concept blocks covered background material used in the Introduction and Discussion, comprising broad terms relating to psychological stress, anxiety, depression, burnout and cortisol on the one hand, and to sleep and injury on the other. These blocks were searched mainly in PubMed; they were deliberately non-specific and returned a total of 65,468 records. They were not screened exhaustively; background and contextual literature was identified purposively, with priority given to consensus statements, systematic reviews and meta-analyses. Their hit counts are therefore reported for transparency but are not included in the screening counts above. In total, 161 publications were included in this review.

Because the present work is a narrative rather than a systematic review, screening was conducted iteratively, and the number of records excluded at each individual stage was not logged prospectively. The record counts reported above were obtained by re-running the complete set of database queries. The number of full texts assessed for eligibility cannot be reconstructed retrospectively and is therefore not reported; a formal PRISMA flow diagram is not presented. We acknowledge this as a limitation of the narrative approach.

## 3. Results

### 3.1. Psychological Interventions and Relaxation Techniques

Psychological interventions and relaxation techniques constitute a critical component of comprehensive sports preparation. Substantial scientific evidence supports the effectiveness of mindfulness, breathing techniques, and imagery training. The following section examines the impact of these methods from a cross-disciplinary perspective and highlights the increasing integration of relaxation and psychological approaches into athletes’ daily training routines [12].

#### 3.1.1. Self-Talk (ST)

The idea behind strategies that involve self-talk is that cognitive processes, for example thoughts, have a significant effect on behavioural outcomes [13]. In the field of sport psychology, self-talk has become an important psychological technique intended to improve athletic performance [14], emotional regulation [15], attentional control [16], and motivation [17].

ST is most often described as a type of intrapersonal communication in which the person who sends the message is the same as the one who receives it [18]. It usually involves self-directed verbalisations, ranging from single words, short comments, and commands to more detailed internal statements, and such communication does not require reciprocal interaction. Brinthaupt et al. further developed the functional approach to ST by identifying a few main functions of self-talk, such as self-criticism, self-reinforcement, self-management and social assessment [19]. In sporting situations, ST can have both instructional and motivational roles. Instructional self-talk is generally used to direct attention to task-relevant cues and to improve technical performance (for example, ‘focus on your breathing’) [20].

On the other hand, motivational self-talk is generally used to boost confidence, perseverance and emotional control (for example, ‘stay calm’) [20]. Recent research has also pointed out the importance of grammatical form in self-talk interventions. More specifically, the use of second-person self-talk (for instance, ‘you can do it’) and mentioning one’s own name have been linked to better self-regulation and performance results than first-person formulations, possibly because of the greater psychological distance and increased objectivity experienced in stressful situations [21]. Because it is easy to access, adaptable and has been shown to be effective, self-talk has become one of the most used psychological techniques among both top-level and recreational athletes. Modern-day interventions often include structured self-talk strategies to optimise performance when under pressure, to regulate emotions and to support coping mechanisms during training and competition. Self-talk is a very effective way of improving athletic performance whether in individual or team sports [22]. In a study carried out by Bellomo et al., it was discovered that instructional self-talk led to an improvement in the putting technique of beginner golfers, as shown by a reduction in lateral clubhead acceleration [23]. Yet, no significant differences in putting accuracy were found. In a study carried out by De Matos et al. [24], motivational self-talk increased the swimming endurance of triathletes.

A different study involving rugby players showed that motivational self-talk had an effect on both the performance and the kinematics of the vertical jump, allowing the athletes to attain greater heights [25]. The beneficial effect of long-term self-talk on athletes’ performance was clear whether the situation involved isolated movements or skills or in terms of the athlete’s overall effectiveness in their particular sport. In the research carried out by Walter et al., the athletes who had completed an 8-week self-talk course were considered more effective by their coaches than those who had not taken part or had only completed a short-term course [22]. Moreover, it has been found that self-talk is advantageous in aiming-related sports such as basketball [26] and shooting [17]. Instructional self-talk was moderately linked to a longer ‘quiet-eye’ duration as well as to the onset and offset of this duration, and to a longer duration of the longest fixation during successful free throws; it was also linked to a lower fixation frequency in both successful and unsuccessful shots [26]. Self-talk also has an impact on performance-related aspects of mental health, such as emotions, cognitive processes and personality traits. Using self-talk enables athletes to build up self-confidence both during training and in competition, especially when it is combined with other psychological skill training methods such as mental imagery [27,28]. In the study by Park et al. [17], shooting athletes who used self-talk had higher levels of intrinsic motivation and enjoyed the sport more, which could in turn lead to improved performance. Anxiety and mental fatigue resulting from prolonged physical exertion and competition are important factors that hinder athletic performance [29,30]. Motivational self-talk reduced cognitive anxiety in a group of tennis players [15]. Among cyclists, it decreased the subjective feeling of fatigue while at the same time increasing the time to exhaustion [31]. Negative self-talk caused by the stress of physical exertion and competition raises anxiety levels and hampers performance [32,33]. It is a difficult but effective approach for athletes to develop and improve their self-talk in order to overcome negative self-talk [34]. New and unorthodox forms of self-talk in sport, such as swearing, need to be investigated further; however, they might be useful tools for improving performance and supporting the mental health of athletes [35].

#### 3.1.2. Imagery and Visualisation

Imagery involves a multimodal simulation that allows an athlete to experience sensory input even in the absence of external sensory stimulation. This simulation produces a mental situation that can be experienced through the five senses and is intended to enhance an athlete’s performance. Among the psychological techniques, imagery training is of great importance. Regular practice with imagery improves reaction speed and the development of motor and technical skills. It has been found, as is the case with other methods, to boost athletes’ self-confidence and to lessen their pre-race anxiety, especially in sports that demand concentration and precision [36,37]. There is a particular form of imagery called motor imagery. Motor imagery can be described as a dynamic state in which the representations of a particular motor action are internally rehearsed in working memory without any overt motor output [38]. A systematic review and meta-analysis of 86 studies involving 3593 athletes, conducted by Liu et al., demonstrates the positive effect of imagery practice on athletic performance and recommends an optimal dosage for this intervention. The researchers concluded that each session of imagery practice should last 10 min, be carried out three times a week, and continue for more than 100 days to obtain the desired results. However, different kinds of motor performance may call for specific dosages. Within the performance model, imagery practice led to improvements in muscular strength, agility and performance, especially in tennis and football. The intervention produced better results in healthy male amateur athletes who had no previous psychological training [36].

Moreover, this study indicates that when imagery training is combined with other kinds of psychological training—such as ST, video observation, or virtual reality (VR)—the results are better than those obtained with imagery practice alone [36]. An earlier meta-analysis carried out by Simonsmeier et al. (which involved 55 studies and 1438 participants) also shows that imagery interventions had a statistically significant, moderate positive effect on performance when compared to the no-treatment control conditions (d = 0.431, 95% CI [0.298, 0.563]). One of the main findings of this study was that the effect of imagery was dosage-specific, meaning that the more imagery sessions one has, the more effective the imagery interventions become [39].

It is necessary to realise that guided imagery programmes not only improve athletes’ performance but also their imagery skills. A quantitative, survey-based and experimental study carried out by Volgemute et al. was designed to find out if different imagery ability patterns could distinguish athletes at various levels of achievement by means of the Sport Imagery Ability Questionnaire (SIAQ). The researchers divided the 500 athletes into four groups according to both their imagery skills and sporting success. The athletes who were highly achieving and belonged to Cluster 1 had strong imagery abilities, while those who were less achieving and were in Cluster 3 showed weaker imagery abilities. The athletes with average sporting success were split into two groups: Cluster 2, in which the imagery abilities were low, and Cluster 4, in which both the level of achievement and the imagery skills were average. This grouping allowed the researchers to create individualised psychological skills interventions to meet the athletes’ requirements and take account of their imagery profiles. In the experimental phase of the study, nine alpine skiers from Cluster 2 received a six-month guided imagery intervention in addition to their normal training. The results showed clearly that the guided imagery intervention had a significant effect on all imagery abilities except mastery imagery, as found in the post-intervention assessment using the SIAQ [40].

Athletic performance in the control training sessions also exhibited significant improvements. Even though the number of people taking part in the experimental section of the study was small, it does provide a basis for future research concerning the role of imagery abilities in athletes’ performance and for the development of individualised imagery interventions that are appropriate to athletes’ needs and resources [40]. In the literature, the terms ‘imagery’ and ‘visualisation’ are generally used interchangeably. Nevertheless, some authors keep ‘imagery’ for multimodal simulation which involves several sensory modalities and reserve ‘visualisation’ for just the visual aspect. To maintain consistency, this review makes use of the term ‘imagery’ throughout [41]. More experienced athletes in their respective disciplines can use imagery more effectively and acquire expertise in it over time [42]. It has been suggested that technological innovations which support imagery are more attractive to younger athletes, while older athletes tend to prefer traditional methods such as breathing exercises and meditation [41].

#### 3.1.3. Relaxation Techniques (RTs)

RTs consist of several methods that aid in the reduction in both psychological and physiological tension by means of techniques that indirectly boost parasympathetic activity [43]. Intentional regulation of physiological reactions can be obtained by using breath control, regulating muscle tension, focusing attention, or carrying out visualisation exercises. The RTs most frequently employed are deep breathing, meditation, yoga, tai chi, Jacobson’s Progressive Muscle Relaxation (PMR), Schultz’s autogenic training, and mindfulness techniques. Since professional athletes are a group that is especially susceptible to chronic stress, they often suffer from a prolonged activation of the sympathetic nervous system. This is the reason why RTs have been widely adopted among them. This kind of approach helps to control pre-competition arousal, improve emotional regulation, increase concentration and mental resilience, speed up post-exercise recovery, and thus contribute to overall athletic performance [44]. RTs are becoming an integral part of complete athlete preparation and their effects in various areas of sport are being widely examined. A few studies show that mindfulness-based interventions are among the most used techniques in sport [45]. However, other methods such as breathing exercises, imagery training, PMR, and emotional self-regulation can also offer valuable support [12]. Regular practice of mindfulness may lead to improvements in attention, emotion regulation, stress control, mental resilience and athletic performance, both for amateur and professional athletes [12,46]. A decrease in pre-race anxiety and greater emotional stability have also been noted in athletes [47]. The clear effectiveness of mindfulness was associated with meeting two conditions: consistent and long-term practice, along with training sessions [46]. In turn, controlled breathing exercises have been found to regulate the autonomic nervous system by increasing heart rate variability (HRV). Reduced psychophysical tension and better stress management in athletes have also been observed. Particularly positive effects were seen with slow-paced breathing, as this improves concentration and helps to regulate pre-exercise arousal [48]. Interestingly, different breathing techniques can have different physiological effects. Calm and slow breathing reduces tension and anxiety, while more stimulating forms of breathing can increase the willingness to exercise and the arousal levels needed for dynamic sports [49,50]. It has been found that breathing techniques can support post-exercise recovery, improve movement control, and enhance performance in sports that demand high precision and concentration [51].

Since yoga programmes are intended to improve psychosocial function, they might also help in preventing sports injuries. A meta-analysis indicated that interventions designed to reduce stress and enhance skills in stress management can lower the risk of injury among athletes [52]. A small, controlled study involving male university athletes over a 10-week period found that twice-weekly yoga sessions led to improvements in certain flexibility and static balance measures; yet it did not look at the occurrence of injuries, so it cannot be said that an injury-prevention effect has been established [53]. In addition to their effects relating to injury, randomised trials have shown that yoga-based interventions can reduce stress and improve mood, mainly among people who are not athletes [54]. Comparable mental health and quality-of-life benefits have also been recorded in clinical settings, for example among women undergoing treatment for breast cancer [55]. It has not, however, been directly tested whether these effects also apply to athletes’ recovery and sleep quality.

PMR is a type of relaxation technique; in this technique, participants systematically tighten and then relax particular muscle groups to decrease both physical tension and cognitive anxiety. Focusing on the difference between muscular contraction and relaxation increases participants’ awareness of their bodily sensations and helps reduce their stress responses [56].

The findings of randomised controlled trials and quasi-experimental studies indicate that PMR is effective at reducing stress, anxiety and depression among adult members of the general population. In their systematic review, Khir et al. found 24 studies which supported the positive effect of PMR on stress, 21 on anxiety and 11 on depression. PMR might be more effective when used in combination with other psychological interventions [57]. Moreover, PMR has been looked at as a possible supplementary technique to help cancer patients, as well as those with mental health problems and those affected by COVID-19 [58,59,60,61]. It seems that PMR could also help to regulate mood in young athletes by lowering levels of confusion, depression, fatigue and tension [62]. A study by Battaglini et al., which involved 59 basketball players aged 14 to 19, showed that twelve PMR sessions—each lasting 30 to 40 min and given 1 to 2 times a week after training—led to a significant reduction in cognitive anxiety, as measured by the Competitive State Anxiety Inventory-2 (CSAI-2), and in sport-related stress, as measured by the Recovery-Stress Questionnaire for Athletes (RESTQ-Sport) [63].

There were no notable changes in somatic anxiety or self-confidence, which indicates that PMR mainly affects the psychological rather than the physiological aspects of anxiety. Moreover, PMR was found to be linked with a lowering of heart rate over several sessions, suggesting that it could be of use in facilitating recovery and in controlling the body’s physiological reaction to anxiety [63]. It has been demonstrated that relaxation training can enhance self-confidence prior to a competition, especially during the first and second weeks of the intervention and might be positively linked to better athletic performance [64]. A randomised controlled study carried out by Abdelkefi and Jarraya examined the effect of PMR on affective well-being, cognitive function, and aggressive behaviour in female athletes between the ages of 18 and 22 during their menstruation (*n* = 45). This study compared eight 30 min sessions of PMR with eight 30 min sessions of deep breathing and included a control group. The results showed that PMR could improve mood and decrease anxiety; no difference was found regarding aggressive behaviour or cognitive variables [65]. In the study by Vento et al., a single 20 min session of PMR did not result in greater improvements in the positive emotional state or in fatigue than 20 min of quiet rest. The only significant difference between the groups was with respect to perceived stress, the group that rested quietly showing a greater reduction than the PMR group. Both conditions produced significant improvements from before to after in the positive emotional state, stress and fatigue. However, since the study used a quasi-experimental design and lacked a no-treatment control group, the within-group changes cannot be attributed to the rest conditions themselves [66].

Moreover, the small number of participants (*n* = 30) means that the study had limited ability to detect differences between the groups, and the fact that the research was carried out in a controlled laboratory environment limits the extent to which the results can be applied to real-world training and competitive situations [66]. It was shown that among young athletes aged 13 to 14 years (*n* = 26), those who received PMR training for 16 muscle groups had significantly shorter reaction times and lower heart rates than the group that followed a protocol involving only 7 muscle groups. Both groups exhibited significant reductions in heart rate and choice reaction time [67]. Hashim and Yusof carried out a comparison between PMR and autogenic relaxation (AGR) among sixteen adolescent football players. During the testing sessions, levels of confusion, depression, fatigue and tension all decreased, with no significant interaction between group and session; however, since there was no control group, the pre-post changes cannot be attributed to the relaxation techniques themselves [62].

Current evidence indicates that progressive muscle relaxation (PMR) is effective in reducing cognitive anxiety, particularly in team sports such as basketball, where pre-competition stress is prevalent. However, the existing research base remains limited, and further studies are required to clarify the extent to which PMR influences athletic performance and psychological well-being.

### 3.2. Psychological Stress Assessment Tools

Another question that deserves to be investigated is whether psychological stress assessment instruments can predict how athletes will respond to competitive stress—and, if they can, which of these tools is the most effective. The CSAI-2, and especially its revised form (CSAI-2R), has been extensively used and assessed in several different groups of athletes and in various language versions [68,69,70,71]. Tang and Lim point out that research into mental training in the West started early, has a well-established theoretical framework, is marked by many empirical studies (particularly randomised controlled trials), and is characterised by interdisciplinary cooperation. In their paper they also mention some limitations, for example the fact that conclusions are mainly based on short-term mental training studies, a heavy reliance on questionnaires and self-reports (which are prone to bias), and the fact that the research has mainly focused on professional athletes, thus restricting the generalisation of the results to a wider population [72]. The IOC has so far published several tools to aid in the early identification of mental health problems among athletes [11]. Regarding tools for assessing psychological stress, especially in the context of competitions, the body of evidence is limited, usually involving small sample sizes, and there are very few direct prospective studies. Nevertheless, mood states, as measured by the Profile of Mood States (POMS) questionnaire, have been widely researched in sports psychology. In the 1980s, Morgan suggested using this questionnaire to differentiate between more and less successful athletes. The high-performing athletes had a particular mood profile, which Morgan referred to as the ‘iceberg profile’. When plotted on a graph the mood distribution looks like an iceberg, with vigour standing well above the baseline while the other mood characteristics (including the negative ones) are below it [73,74]. Lochbaum et al. found that Morgan’s mental health model, with the exception of anger, still served as a valid framework for predicting athletic performance. Depression, confusion and vigour can be used as predictors before a competition, successful athletes having lower levels of depression and confusion and higher levels of vigour. Total mood disturbance (TMD), tension and fatigue only become statistically significant after adjusting for publication bias, TMD showing a large effect size. The authors point out that anger does not fit into Morgan’s model since it can be advantageous in short-duration sports such as judo, karate and weightlifting [75]. Higher levels of stress and a lower sense of well-being were found to be significant predictors of poorer athletic performance. In the study by Blume and Wolfarth, those athletes who reported a greater degree of performance improvement had higher subjective well-being and lower stress than those who showed less improvement. Interestingly, both groups of athletes trained for the same number of hours per week [76]. A correlation was discovered between cognitive anxiety and performance in competitive sports. High levels of cognitive anxiety had a negative effect on athletic performance, the connection being stronger among individual athletes and women. Regarding somatic anxiety, moderate arousal seems to improve performance, while too little or too much arousal impairs it. The authors described this relationship as inverted U-shaped [77]. These findings are not necessarily in conflict; the contribution of cognitive and somatic anxiety seems to depend on the nature of the task. Mabweazara et al. found that somatic anxiety was the strongest predictor of athletic performance, noting that both cognitive and somatic anxiety influenced performance. The authors stress that the study was carried out among 50-m swimmers and conclude that somatic anxiety has a greater effect in sprint competitions, where excessive arousal or bodily tension (excessive stress reactions) can impair athletic performance [78].

It is particularly important to assess athletes’ stress responses in the early stages of competitive sport, as this information can inform the development of targeted interventions to improve performance outcomes among young athletes.

#### 3.2.1. Athlete Psychological Strain Questionnaire (APSQ)

The APSQ is a well-known questionnaire used for evaluating the symptoms of psychological strain among athletes and was developed by Rice et al. A validation study was carried out on 1007 male elite professional athletes from three different sports in Australia (Australian rules football, cricket and football), these athletes making up 78.6 per cent of the players contracted in those sports. The APSQ includes 10 items and addresses three major psycho-emotional areas: difficulties with self-regulation, worries concerning sporting performance, and the ability to cope with external pressure. The results showed that the tool had good reliability and validity, allowing the authors to state that the APSQ can be used as a screening test for the early identification of psycho-emotional problems in elite male athletes. In a later study, Rice et al. showed that the questionnaire can also be applied to women. Moreover, three cut-off points were set, corresponding to different degrees of symptom severity: moderate, high and very high risk of mental health issues. This allows for a more accurate identification of those athletes who need further evaluation or psychological assistance [79,80]. The APSQ is widely employed when assessing elite athletes and is included in the IOC protocol, as will be discussed later in this paper. In their scoping review, Dragoiu et al. stated that the currently validated versions of the APSQ are the Arabic, Croatian, Spanish, Indonesian, Japanese, Persian, Polish and Turkish adaptations. The authors also recommend that the APSQ could be useful as a triage tool, even though it should not be considered a standalone diagnostic instrument. The APSQ showed good screening validity with respect to anxiety, depression and thoughts of self-harm, but had only limited validity in the case of alcohol misuse, sleep disturbances and disordered eating [81]. It is also important to note that the APSQ is still undergoing continuous validation. In March 2026, Dragoiu et al. assessed the Romanian version of the APSQ and obtained results like those reported in the previous study [82].

The APSQ is also used as the first-stage triage tool (Step 1) in the SMHAT-1, which is carried out before any disorder-specific screening. The SMHAT-1 is a standardised assessment procedure developed by the IOC Mental Health Working Group and published by Gouttebarge et al., with the aim of identifying elite athletes who are at risk of, or who are already experiencing, mental health symptoms and disorders. The athletes evaluate 10 items in relation to the previous 30 days, and their total score varies between 10 and 50 points. If the total score is between 10 and 16, no immediate action is needed other than routine re-triage; however, if the score is 17 or above, Step 2 of the protocol is initiated and the athlete then completes six standardised screening tools which look at anxiety, depression, sleep disturbance, alcohol misuse, drug misuse and disordered eating. It is important to note that the APSQ is also employed at a later stage of the SMHAT-1 as a monitoring tool: after having undergone the brief interventions in Step 3a (for example, psychoeducation, mindfulness training or mental skills training), the athlete is reassessed using the APSQ and if their score remains at 17 or above, they are referred to a comprehensive clinical evaluation (Step 3b). In line with IOC recommendations, APSQ-based triage should ideally take place during the pre-competition period, as well as midway through and at the end of the season, and also at any time when a significant event happens in an athlete’s career, such as an injury, surgery, an unexplained decline in athletic performance, a major competition, suspected harassment or abuse, or the end of an athletic career. Thus, within the SMHAT-1 framework, the APSQ functions not as a diagnostic tool but rather as a screening and monitoring instrument to indicate whether further detailed screening and a clinical evaluation are necessary [11].

#### 3.2.2. Competitive State Anxiety Inventory-2 (CSAI-2)

The CSAI-2 is one of the most used questionnaires for measuring stress and anxiety among athletes. It was developed by Martens et al. as a multidimensional extension of the original CSAI and includes 27 items which evaluate cognitive anxiety, somatic anxiety and self-confidence [68]. However, further confirmatory factor analyses failed to support the original 27-item measurement model since a few of the items loaded on more than one factor. Cox et al. therefore eliminated ten items that showed cross-loadings, leading to a revised 17-item version (CSAI-2R; 5 items for cognitive anxiety, 7 for somatic anxiety and 5 for self-confidence) that preserved the three-factor structure and showed a good fit with the data [69]. Lundqvist and Hassmén, who were also worried about the reliability of the original CSAI-2, carried out a study in which they compared the original and the modified scales in Sweden. By means of confirmatory factor analysis, the research team found that the CSAI-2R model yields more reliable results than the CSAI-2 [70]. A different group of researchers, headed by Martinent et al., carried out a validation study of the French version of the CSAI-2R, involving 642 men and women who took part in various types of sport and with a particular focus on the scales relating to direction—defined as the interpretation of symptoms associated with competitive anxiety—and frequency—defined as the amount of time spent attending to anxiety symptoms before competition. The results of this study showed that the CSAI-2R, including the direction and frequency scales, is a reliable instrument for assessing stress and anxiety in competitive sports [71]. A meta-analysis of controlled trials found that the pooled effects were numerically greater in the studies that used the CSAI-2 than in those that used the SCAT (Sport Competition Anxiety Test) or the SAS-2 (Sport Anxiety Scale-2). Although the authors regarded this as evidence that the CSAI-2 was more sensitive to change, the basis for this conclusion was observational subgroup comparisons rather than a direct psychometric comparison of the instruments. The CSAI-2 subgroup consisted of 17 heterogeneous studies, while the SCAT and SAS-2 subgroups included only three and two studies respectively; in addition, the analysis did not include a formal test of the differences between these subgroups [83].

#### 3.2.3. Sport Anxiety Scale (SAS)

The desire to create a multidimensional research tool appropriate for use in the ordinary work of sports psychologists and capable of telling the difference between the somatic and cognitive aspects of anxiety resulted in the development of the SAS [84]. The SAS comprises 21 items and is divided into three subscales which assess somatic anxiety, worry and concentration disruption. In the Chinese-language validation of the SAS-2, each of the subscales—somatic anxiety, worry and concentration disruption—was positively related to each dimension of the Athlete Burnout Questionnaire [85] and to the STAI (State-Trait Anxiety Inventory) scores; concentration disruption also acted as a mediator of the relationship between trait anxiety and burnout [86]. Although the original scale proved versatile in assessing anxiety among adult athletes [87], it did not produce reliable results when used with children. In the field study carried out by Smith et al. [88], sport-specific anxiety scales were given to children aged 10 to 12. In these child groups, the three-factor structure of the original SAS could not be replicated, and several items gave conflicting factor loadings even in adult samples [89]. The explanations given for this were either that the questions had an excessively complicated structure, making them hard for younger children to understand, or that the children’s limited emotional self-awareness impeded their ability to differentiate between these three components of anxiety. However, Smith et al. found that children as young as nine years old were able to understand the difference between somatic and cognitive anxiety. For this reason, the researchers developed a new version of the scale, the SAS-2, which kept the three-factor structure but used more understandable questions [89]. As the validation study showed, the SAS-2 not only widened its application to younger children but also became more appropriate for older athletes. A study involving 519 Polish athletes showed that the SAS-2 is a reliable method for assessing anxiety in both professional and recreational sports [90]. Moreover, the SAS-2 continues to be a useful tool for sport psychologists, allowing them to measure the levels of anxiety and stress in athletes and to predict their behaviour in competition [91].

#### 3.2.4. Sport Competition Anxiety Test (SCAT)

The SCAT, which Martens [92] developed, was created to evaluate competitive trait anxiety in athletes confronted with competitive situations and is set out in full in the compendium subsequently produced by Martens, Vealey and Burton [93]. Competitive trait anxiety (A-trait) involves a person’s tendency to view objectively harmless situations as threatening and to react to them by entering a state of anxiety (A-state) that is of an intensity greater than that warranted by the seriousness of the situation [94]. Sports coaches can observe their athletes before competitions and estimate their susceptibility to experiencing anxiety. Yet, there is a poor correlation between their assessment of an athlete’s A-trait and the frequency of the athlete’s A-state. In a study involving 136 female basketball players, the SCAT was found to be a more effective means of assessing A-trait than either coaches’ subjective evaluations or other tools designed to measure A-trait [95]. As a result, the SCAT is commonly used as a tool for measuring the effectiveness of psychological interventions, for example self-motivational talks or stress management programmes. It is well known for having a high level of internal consistency [47,96,97].

#### 3.2.5. State-Trait Anxiety Inventory (STAI)

The STAI, which Spielberger developed, is one of the most used tools for measuring anxiety [98]. The original scale contains 40 items, though a more convenient shortened version is also offered [99]. It is an excellent instrument in clinical practice. It can be used to assess anxiety in patients suffering from a variety of mental and physical disorders as well as in athletes during sporting competitions. It was employed in a study carried out by Yamada et al. to look at the effect of state anxiety on the performance of university volleyball players in competition [100], and in another study by García-Gonzálvez et al. to examine the effect of competition on anxiety and heart rate variability in young tennis players [101].

#### 3.2.6. Heidelberg Risk Sport-Specific Stress Test (HRSST)

A way of evaluating people’s reactions to a severe stressor is through the HRSST. In this method, the participants climb a wall that is 12 metres high and then carry out a controlled descent of about 2 to 3 metres. With this test, researchers have examined, together with other variables, the subjective experience of fear in a situation that involves risk. The HRSST could prove to be a useful tool for analysing the way athletes respond to such situations; yet, since the stressor is very specific, its application might be restricted to athletes who take part in sports having a similar level of risk and to occupational groups like emergency and rescue personnel [102].

#### 3.2.7. Trier Social Stress Test (TSST)

A well-known and highly validated psychological paradigm is the TSST, which assesses physiological and psychological responses to psychosocial stress. Unlike the HRSST, the TSST is a social-evaluative stressor involving no physical danger and consists of three main phases: preparation, public speaking, and arithmetic tasks. Depending on the specific TSST protocol, studies have measured subjective parameters, including perceived stress, anxiety and negative affect, as well as physiological markers such as salivary cortisol and, in some cases, adrenocorticotropic hormone (ACTH), growth hormone, prolactin, heart rate, blood pressure and heart rate variability. Campbell and Ehler demonstrated that although the TSST reliably elicits both psychological and psychophysiological stress responses, only approximately 25 per cent of studies reported significant correlations between cortisol responses and measures of perceived emotional stress [103].

#### 3.2.8. The Perceived Stress Reactivity Scale (PSRS)

The Perceived Stress Reactivity Scale is a 23-item questionnaire created by Schlotz et al.; it measures an individual’s stress reactivity and thus the intensity with which that person typically responds to stressful situations in daily life. The scale also enables assessment of individual differences in how people respond [104]. Britton et al. conducted a validation study of the Perceived Stress Reactivity Scale for Adolescent Athletes (PSRS-AA), a modified form of the original PSRS. No significant correlations were discovered between the PSRS-AA scores and physiological variables such as high-frequency heart rate variability. Nevertheless, PSRS-AA scores were linked to the subjective stress response observed during the socially evaluated cold pressor test, indicating that the scale can capture individual differences in perceived stress. This could be of great value in identifying young athletes who need specific psychological support [105,106].

### 3.3. Coping Strategies

The high level of demand in competitive sport subjects athletes to a great deal of stress. Coping is a self-regulatory approach designed for managing stressors. As outlined in the transactional model, this process includes identifying the stressor (primary appraisal) and then choosing a coping strategy (secondary appraisal) [107,108]. Earlier studies have demonstrated that coping is linked to both athletic performance and mental well-being [109]. At first, coping strategies were divided into those that are problem-focused and those that are emotion-focused. Nicholls et al. created a new classification specific to sport based on a three-factor model; this classification splits coping strategies into mastery (those aimed at removing the stressor), internal regulation (those concerned with managing the body’s reaction to stress), and goal withdrawal (those involving avoidance of the stress by giving up the goal). A meta-analysis carried out by the authors of this classification showed that mastery coping was positively correlated with athletic performance, while goal withdrawal was negatively correlated; internal regulation had only negligible negative effects [110]. The ability to combine different coping strategies—coping flexibility—may offer further advantages, as shown by a systematic review of coping among university tennis players carried out by Gao et al. [111].

Several studies have investigated the coping strategies used in various sporting disciplines. Among competitive dancers, mental contrasting and ‘humour’ were found to be the most effective coping methods [112,113]. In the case of volleyball, goal setting and mental preparation were linked to better performance [114], while seeking social support was found to be related to higher levels of perceived stress [115]. Ultra-long-distance runners developed coping strategies that were specific to the kinds of stress they encountered during the run. Functional coping strategies were more frequently used by experienced runners [116]. Dai et al. noted that both perceived social support and perceived ability had a positive effect on the coping of football players [117]. Table tennis players were more inclined to resort to avoidance coping and emotion-focused coping when the level of cognitive anxiety was high [118]. Tennis players who employed strategies such as active planning, cognitive restructuring, emotional calmness and seeking social support experienced greater perceived autonomy and improved psychological well-being [119]. Doron and Martinent found a positive relationship between challenge appraisal, task-oriented coping, positive emotions and performance in elite fencing [120].

Apart from dealing with stress on an individual basis, athletes who take part in team sports also develop collective coping strategies when facing common stressors, for example social pressure, difficulties in their relationships with teammates, problems concerning team performance and organisational problems. Leprince et al. found four types of communal coping strategies among athletes: problem-focused communal efforts, relationship-focused coping, communal management of emotions, and communal goal withdrawal [121]. It is still not clear, however, how communal coping influences athletic performance and so further research is needed [122]. Coping strategies also contribute to the prevention of athlete burnout [123]. In a study by Mei et al., both problem-focused and avoidance-focused coping strategies partly mediated the relationship between resilience and athlete burnout [124]. It is also important to remember that other factors, such as resilience, defence profiles and cognitive appraisal, should likewise be considered when examining the impact of stress and coping on athletic performance [125,126,127].

An overview of the main coping frameworks mentioned above is given in Table 2: the three-factor model, coping flexibility, communal coping, and the modifiers that influence the relationship between stress, coping, and performance. 

**Figure 1:**
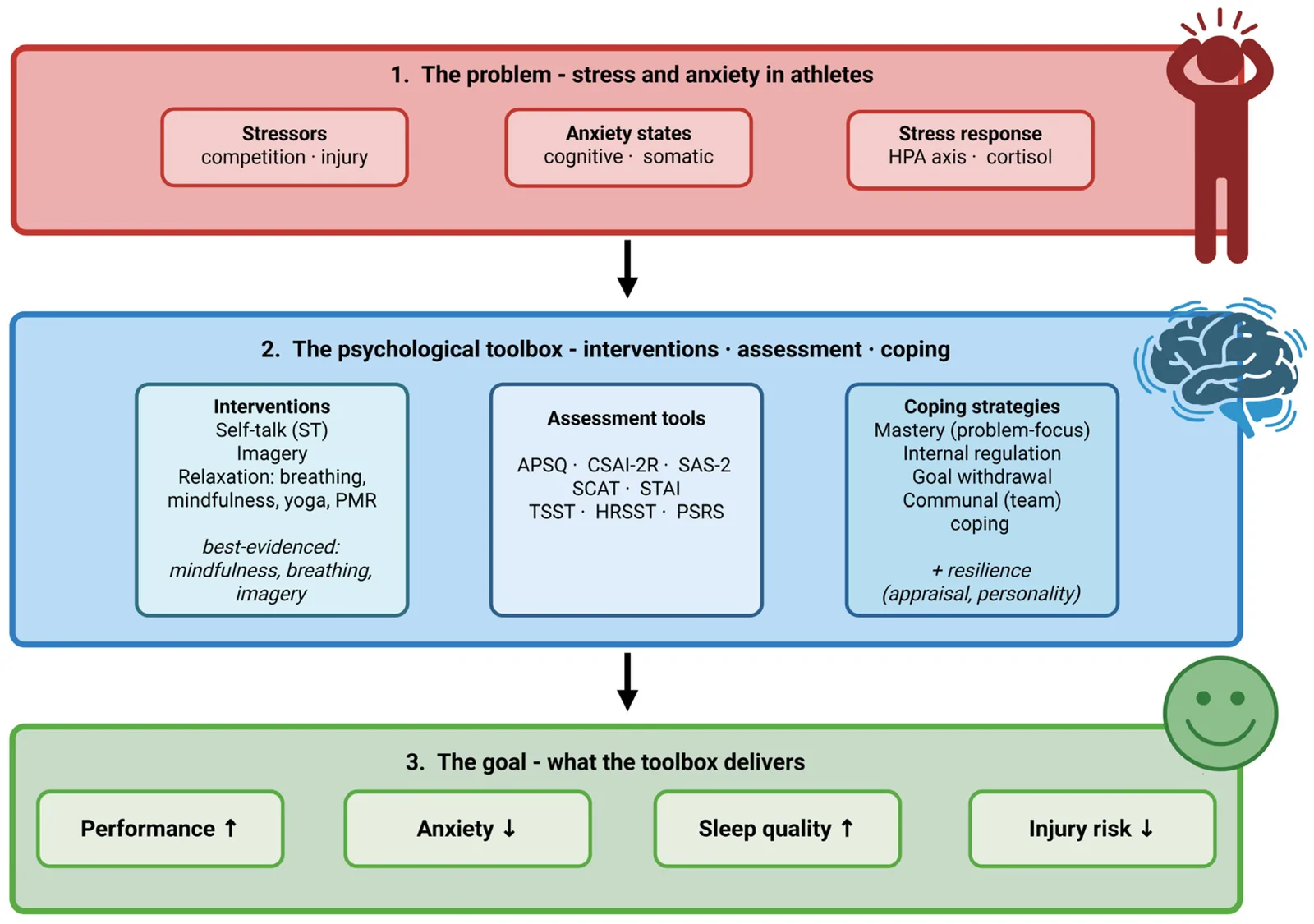
Coping with stress in athletes. Abbreviations: HPA, hypothalamic–pituitary–adrenal (axis); ST, self-talk; PMR, progressive muscle relaxation; APSQ, Athlete Psychological Strain Questionnaire; CSAI-2R, Competitive State Anxiety Inventory-2 Revised; SAS-2, Sport Anxiety Scale-2; SCAT, Sport Competition Anxiety Test; STAI, State-Trait Anxiety Inventory; TSST, Trier Social Stress Test; HRSST, Heidelberg Risk Sport-Specific Stress Test; PSRS, Perceived Stress Reactivity Scale. Black arrows indicate the sequence of the framework, leading from the problem, through the psychological toolbox, to the expected outcomes. Upward arrows (↑) denote an expected increase or improvement, and downward arrows (↓) denote an expected decrease or reduction in a given outcome.

A graphical summary of this review is presented in Figure 1. It illustrates the pathway from the problem, including the main stressors faced by athletes (competition and injury), the resulting cognitive and somatic anxiety states, and the physiological stress response mediated by the HPA axis and cortisol, through the psychological toolbox identified in the included studies, comprising interventions (self-talk, imagery, and relaxation techniques such as breathing exercises, mindfulness, yoga, and progressive muscle relaxation), assessment tools (e.g., APSQ, CSAI-2R, SAS-2, SCAT, STAI), and coping strategies (mastery, internal regulation, goal withdrawal, and communal coping), to the expected outcomes: improved performance and sleep quality, together with reduced anxiety and injury risk.

## 4. Discussion

Sports psychology encompasses both the examination and measurement of stress and the evaluation of methods for managing stressful situations, commonly referred to as coping strategies. Researchers and clinicians in this field investigate the development and effectiveness of coping processes, including the assessment of specific coping strategies and styles.

Psychological stress is a kind of relationship (an interaction) between a person and their environment, and it is not so much the experience itself (the event or situation) that is important as it is the way the individual perceives and interprets it. The transactional approach to stress highlights the importance of a person’s subjective evaluation of an event or situation [128,129]. It is the individual who gives meaning to their experience by judging a particular situation, for instance, as a threat, a cause of harm or a loss, or as a challenge. A transaction can also be seen as a positive or neutral (meaningless) situation, meaning it does not constitute a threat to psychological well-being. The effects of stress are more closely tied to how an individual evaluates an event or situation than to the objective features of the stressor. It should be noted, however, that the transactional approach to stress makes it difficult to measure stress—a question then arises as to how the interaction between an individual and their environment is to be measured [130]. Because of this difficulty, research on stress and coping most often uses scales that measure reactions to specific events. Stress is defined as the physiological and psychological consequences a person experiences in response to events (stressors). Assessment of stress involves evaluating both the immediate effects of the event (such as physiological changes, emotional and cognitive reactions) and the long-term consequences (disorders affecting physical and mental health). The methods employed to assess stress and coping vary according to their psychometric properties. This in turn allows a certain amount of discretion when interpreting the research findings and can therefore result in ambiguity and, at times, even in contradictory conclusions. In this regard, the careful selection of tools for measuring stress and coping is of key importance—the tools should be standardised, adapted in accordance with current guidelines, and have high validity and reliability [130]. The scales described in Section 3 are used to assess the level of stress experienced by athletes. They are intended for research purposes and are used in practice, in screening and preventive studies, and in evaluating the effectiveness of therapeutic interventions. They can be used by specialists (such as psychologists, psychotherapists and doctors) working in the areas of stress, health promotion, health education, prevention and therapy. The use of these tools requires an understanding of the theoretical background, as well as knowledge of the method’s intended purpose and its limitations. When interpreting the results, knowledge of psychology and psychometrics as well as an understanding of the principles for interpreting these methods is essential [130]. The tools described in this article cover a range of dimensions of an individual’s functioning, their personality traits, and the individual aspects of experiencing, perceiving and coping with challenges, as well as psychological and physical strain. The choice of psychometric tool(s) is closely connected with the aim of the study. The responsibility for deciding which tool to use, carrying out the study, correctly interpreting the results, identifying the problem and implementing appropriate forms of intervention lies with the professionals who provide multidimensional support to athletes.

Elite athletes must cope with a particular range of stresses which may raise their risk of mental health problems. The various stressors that elite athletes experience, some of which can be damaging to their mental health, include the psychological effects of physical injuries and overtraining, together with pressure from their surroundings and from the public to improve their performance and get better results [3]. It was also noted that there is a statistically significant correlation between higher levels of life stress and the occurrence of depressive symptoms among elite athletes. Female athletes have more life stressors than male athletes who have a similar level of performance [131]. Research carried out by Chang et al. has shown that athletes have certain risk factors for depression and anxiety when compared to non-athletes [6]. The review by Golding et al. points out that depressive symptoms are very common among high-performance athletes [132]. A comparative meta-analysis carried out by Gorczynski et al. showed the presence of signs and symptoms of depression and anxiety in the group of student-athletes, with females being mainly at greater risk than males [133,134]. Athletes have a particular set of risk factors related to depression that are not usually seen in non-athletes, namely sports injuries, being forced to end their careers, and the expectations regarding performance. As a result, it is essential to carry out early screening and to properly manage depression to achieve better clinical and performance outcomes [6,135].

Stress has always been associated with the risk of injury [6,136], as well as with an athlete’s ability to recover from injury and to return to sport [6]. Gil-Caselles and colleagues found a positive correlation between the number of injuries among triathletes and three factors—stress, anxiety and depression—with stress being the most important of these [137]. It has been demonstrated that an acute stress response impairs both cognitive and physical functioning, causing a shorter attention span, greater distractibility and increased self-consciousness, together with heightened muscle tension and poor motor coordination. The combined effect of these factors leads to a noticeable drop in performance and a greater likelihood of suffering an injury [8]. Li and others showed that athletes who displayed anxiety symptoms before the season had a considerably higher incidence of injuries than those who did not have such symptoms [138]. Just as with stress, sport-related anxiety can increase the risk of injury, slow down and obstruct the rehabilitation process and return to sport, and raise the chance of re-injury during training and competition [5]. Psychological interventions can influence injury risk among athletes [52]. Contreras and colleagues noted that high levels of stress and sport-related anxiety in student-athletes were the most reliable predictors of dysfunctional coping (such as self-distraction, denial, withdrawal from behaviour, self-blame, use of psychoactive substances, and emotional outbursts) [139].

Individual characteristics shape the appraisal of situations and the selection of coping strategies, thereby influencing coping effectiveness. Accurate assessment of circumstances and the implementation of appropriate coping strategies facilitate problem-solving, enhance perceived control, and reduce psychological distress. However, some situations may remain unresolved, leading to increased psychophysiological tension, heightened negative emotions such as anxiety and anger, and reduced perceived control. In these instances, psychological interventions and relaxation techniques may help mitigate the stress response.

Anxiety associated with sport is a negative response generally connected with the stress of taking part in sporting activities [5]. State anxiety is especially frequent among athletes right before a competition, a state which is known as pre-competition anxiety (PCA). PCA usually appears as fear, tension and concern on the part of athletes at the beginning of the sporting event. Objectively, activation of the sympathetic nervous system can be detected. Excessive PCA can be harmful to athletic performance because it leads to a lack of concentration and poor decision-making. The long-term negative consequences of PCA are depression, burnout and early retirement [140,141]. Competitive trait anxiety has been found to be a psychosocial factor that contributes to the risk of musculoskeletal injury in athletes. However, its independent effect seems to be small and is moderated by other factors, such as stress from life events and an individual’s coping resources [142].

Anxiety connected with sport is a complex and multi-dimensional thing; for this reason, researchers concentrate on finding the factors which determine whether it occurs and how intense it is, as well as those which help to reduce it. It has been found, among other things, that the amount of anxiety exhibited by athletes is linked to gender, age and the type of sport (individual or team). Research shows that women have higher levels of general sport-related anxiety [143], greater levels of competitive anxiety [144,145,146,147], higher levels of somatic anxiety [148], and higher levels of the worry-related components of sport anxiety [148,149], as well as higher levels of cognitive, motor, physiological and total anxiety than do male athletes [144]. Athletes who take part in individual sports showed higher levels of competitive anxiety [147], and higher average levels of somatic anxiety [150,151] than those who compete in team sports. Children and adolescents who take up sports appear to follow a similar pattern [152]. Rice et al. found that two factors specific to athletes—existing musculoskeletal injuries and dissatisfaction with their sporting career—are potential risk factors for symptomatic anxiety in elite athletes [153]. Personality is important for achieving high levels of success in sport. Personality traits relate to long-term sporting success, interpersonal relationships, and the mental states of athletes before, during and after competition [154]. Although certain personality traits may help to achieve athletic success, the same traits can also be linked to mental health problems [6]. Personality traits also influence the level of pre-competition anxiety. A person’s level of trait anxiety influences how the situation is perceived, since it is a personality feature that makes a person tend to see a competition as either more or less threatening [155]. Neuroticism has been identified as a personality trait with important consequences for competitive performance in sport. It is defined as a dispositional tendency to experience negative emotions—such as anger, anxiety and depression—and neuroticism indicates a general tendency towards emotional instability and a low tolerance for stress and external stimuli [147]. Personality traits and disorders that are problematic for athletes can most effectively be treated by psychotherapy [6].

Anxiety could potentially act as a risk factor for sleep problems among athletes [153,156]. Cognitive anxiety seems to occur before sleep disturbances when competition is involved. Several psychological factors affect pre-competition anxiety. For example, research carried out in China has discovered strong associations between psychological stress, anxiety, sleep problems and depression [2]. There has always been a connection between sleeping for seven hours or less and an increased risk of injury [157]. Athletes are at a high risk of getting insufficient sleep, having poor sleep quality, experiencing daytime sleepiness, feeling fatigued, maintaining irregular sleep schedules, and suffering from sleep and circadian rhythm disorders [158]. Also, sleep is closely linked to major mental health problems such as depression, anxiety and stress amongst elite athletes [158,159,160].

Sleep problems may also raise the chance of suffering concussions, musculoskeletal injuries and pain, and can impede recovery after an injury [157,158,160,161]. Athletic performance decreases when circadian rhythms are disturbed; furthermore, continuous dysregulation might also lead to neurodegeneration and mental health problems [8].

## 5. Limitations

The strength of the conclusions drawn from the primary literature is restricted by several factors. First, a great many of the intervention studies were carried out with small samples—in most cases fewer than 60 and in some instances fewer than 30—which reduces statistical power and raises the chance of both false-positive and false-negative results. Second, the samples were often taken from narrow groups—such as a single sport, a single sex, or a limited age group (usually adolescents or young adults)—thus limiting the ability to generalise the findings to elite, mixed-sex, or older athletic populations. Third, the study designs varied greatly, including randomised controlled trials, quasi-experimental pre-post designs, cross-sectional and psychometric validation studies, and the outcomes were mainly self-reported, leading to common-method variance and social desirability bias. Fourth, the intervention periods were generally short and follow-up was seldom reported, so it is not clear how long the observed effects last. Finally, most of the intervention findings are based on single studies that have not been independently replicated; in cases where robust replication does take place (for example, in the APSQ validation programme), the evidence is therefore stronger.

Of the various interventions examined, imagery and visualisation show the most consistent and well-replicated form of support, with self-talk and mindfulness-based breathing coming next. The evidence for the use of PMR among athletes is encouraging but still in its early stages because it is based on small and diverse trials. As for assessment tools, the APSQ and the CSAI-2R are the ones that have been validated the most and have been replicated most frequently. Hence, the clinical implications outlined in this review should be regarded as provisional advice of only low to moderate certainty, until they have been confirmed by the results of adequately powered, pre-registered, and, where possible, multi-site randomised controlled trials that provide effect sizes, measures of heterogeneity, and long-term follow-up.

Apart from the limitations of the main evidence, this review also has some inherent restrictions. Since it is a narrative and purposive review and not a systematic one, the studies were chosen to represent the evidence base rather than being selected according to a comprehensive and pre-registered protocol. It therefore cannot be ruled out that there was selection bias, and no formal evaluation of the risk of bias or of the certainty of the evidence was carried out. For screening, only titles and abstracts in English were accepted, which may have excluded relevant work published without these elements; full texts in languages other than English or Polish were assessed using machine translation, which could have introduced minor inaccuracies in data extraction. As no quantitative synthesis was conducted, there are no pooled effect estimates or formal tests of heterogeneity for the interventions in question. It follows that some of the recommendations should be seen as directionally supported and hypothesis-generating rather than as having high certainty and being quantitatively established effects.

## 6. Conclusions

Professional athletes encounter a range of physical and emotional challenges, with stress and anxiety frequently contributing to injuries, depression, and insomnia. Given their heightened risk of both acute and chronic stress, comprehensive psychological support is warranted. Effective care should include regular evaluation and monitoring of stress, alongside targeted psychological interventions that promote adaptive coping strategies. Mental skills and psychological training enable athletes to utilise their internal resources in demanding situations. In clinical practice, a personalised approach is recommended, employing a variety of interventions tailored to the athlete’s specific needs, preferences, and resources.

## Figures and Tables

**Table 1:** A summary of the recommendations on stress management put forward by the IOC for professional athletes.

No.	Recommendation	Key Actions in Practice
1	Mental health literacy and psychoeducation	Teach athletes and staff to recognise signs of stress Develop mental skills (mindfulness, emotion regulation, reframing) Normalise seeking help to reduce stigma
2	Supportive and safe environment	Reduce stressors: excessive travel, overloaded schedules, poor recovery Safeguarding against abuse and harassment
3	Qualified mental health professionals	Confidential, stigma-free, preventive and interventional care Standardised tools (e.g., SMHAT-1) for early detection
4	Interventions during competition	“Mental-fitness”/recovery spaces and on-site support Mental Health Emergency Action Plans
5	Post-competition support	Managing the “post-competition crash” Monitoring and support during reintegration
6	Monitoring of psychological load	Self-report well-being questionnaires: assess mental fatigue Integrate psychological data with physical load monitoring
7	Individualised approach	Tailor strategies to personality, sport, age, career stage and culture
8	Integration into sports medicine	Treat psychological stress as a physical injury Joint psychosocial team assessment; athlete-centred care
9	Emergency preparedness	Action plan + rapid access to trained staff Escalation protocols for panic, distress or breakdown
10	Dual career and work–life balance	Support for education, vocational training and personal development Extra care during transitions (e.g., retirement)
11	Sleep and recovery	Protect sleep from travel/jet lag Structured recovery and sleep optimisation

**Table 2:** The three-factor model of coping strategies (Nicholls et al.), together with coping flexibility, communal coping, and modifiers of the stress-coping-performance relationship.

Category/Model	Description	Relationship to Performance
Mastery coping	Eliminating or tackling the stressor (problem-focused)	Positively correlated with performance
Internal regulation	Managing stress-induced reactions and emotions	Negligible (slightly negative) effect
Withdrawal from the goal	Avoiding stress by abandoning the goal	Negatively associated with performance
Coping flexibility	Combining or switching strategies to suit the situation	Additional benefits (e.g., in university tennis players)
Communal (team) coping	Shared management of team stressors: problem-focused communal efforts, relationship-focused communal emotion management, communal goal withdrawal	Effect still unclear—requires further research
Modifiers	Resilience, defence mechanisms and cognitive appraisal also influence coping	Should be considered when analysing the relationship between stress and performance

## Data Availability

No new data were created or analysed in this study. Data sharing does not apply to this article.

## Abbreviations

The following abbreviations are used in this manuscript:

ACTH	adrenocorticotropic hormone
AGR	autogenic relaxation
APSQ	Athlete Psychological Strain Questionnaire
A-state	state anxiety
A-trait	trait anxiety
CI	confidence interval
CSAI	Competitive State Anxiety Inventory
CSAI-2	Competitive State Anxiety Inventory-2
CSAI-2R	Revised Competitive State Anxiety Inventory-2
HPA	hypothalamic–pituitary–adrenal
HRSST	Heidelberg Risk Sport-Specific Stress Test
HRV	heart rate variability
IOC	International Olympic Committee
PCA	pre-competition anxiety
PMR	Progressive Muscle Relaxation
POMS	Profile of Mood States
PRISMA	Preferred Reporting Items for Systematic Reviews and Meta-Analyses
PSRS	Perceived Stress Reactivity Scale
PSRS-AA	Perceived Stress Reactivity Scale for Adolescent Athletes
RCTs	randomised controlled trials
RESTQ-Sport	Recovery-Stress Questionnaire for Athletes
RTs	Relaxation Techniques
SAS	Sport Anxiety Scale
SAS-2	Sport Anxiety Scale-2
SCAT	Sport Competition Anxiety Test
SIAQ	Sport Imagery Ability Questionnaire
SMHAT-1	Sport Mental Health Assessment Tool 1
ST	Self-talk
STAI	State-Trait Anxiety Inventory
TMD	Total Mood Disturbance
TSST	Trier Social Stress Test
VR	Virtual Reality
